# Duration of night sleep and cognitive performance of community older adults*

**DOI:** 10.1590/1518-8345.4269.3439

**Published:** 2021-06-28

**Authors:** Élen dos Santos Alves, Sofia Cristina Iost Pavarini, Bruna Moretti Luchesi, Ana Carolina Ottaviani, Juliana de Fátima Zacarin Cardoso, Keika Inouye

**Affiliations:** 1 Universidade Federal de São Carlos, Programa de Pós-Graduação em Enfermagem, São Carlos, SP, Brazil.; 2 Universidade Federal de São Carlos, Programa de Pós-Graduação em Gerontologia, Programa de Pós-Graduação em Enfermagem, São Carlos, SP, Brazil.; 3 Universidade Federal do Mato Grosso do Sul, Departamento de Medicina, Três Lagoas, MS, Brazil.; 4 Universidade Federal de São Carlos, Programa de Pós-Graduação em Enfermagem, São Carlos, SP, Brazil.; 5 Universidade Federal de São Carlos, Programa de Pós-Graduação em Enfermagem, São Carlos, SP, Brazil.; 6 Universidade Federal de São Carlos, Departamento de Gerontologia, Programa de Pós-Graduação em Gerontologia, São Carlos, SP, Brazil.

**Keywords:** Sleep, Cognition, Language, Aged, Health of the Elderly, Geriatrics, Sono, Cognição, Linguagem, Idoso, Saúde do Idoso, Geriatria, Sueño, Cognición, Lenguaje, Anciano, Salud del Anciano, Geriatría

## Abstract

**Objective:**

to analyze the relationship between the duration of self-reported night sleep and the cognitive performance of older adults.

**Method:**

the sample consisted of 156 older adults registered in Family Health Units (FHUs) in a city of São Paulo, divided into quartiles according to the duration of night sleep. Data collection was performed using a characterization questionnaire, Addenbrooke’s Cognitive Exam – Revised (ACE-R) and Pittsburgh Sleep Quality Index (PSQI). Descriptive, comparative and correlational statistical analyses were performed.

**Results:**

the older adults obtained a mean of 61.94 points in ACE-R and 55.1% presented good sleep quality. Comparative analyses showed differences between the groups only in the cognitive domain of verbal fluency (p=0.018). The *post-hoc* analyses showed that older adults who slept more hours, a mean of 8.85 hours (Q_1_), had lower scores when compared to those who slept a mean of 6.11 hours (Q_3_) (p=0.004) and of 4.52 hours (Q_4_) (p=0.045). The adjusted model with application of the stepwise method showed a relationship between the independent variables of schooling and sleep duration and the domain verbal fluency.

**Conclusion:**

it is concluded that sleep duration is related to the verbal fluency cognitive domain.

## Abstract (pt)

**Objetivo:**

analisar a relação entre a duração do sono noturno autorrelatada e o desempenho cognitivo de idosos.

**Método:**

a amostra foi constituída por 156 idosos cadastrados em Unidades de Saúde da Família (USF) de um munícipio paulista divididos em quartis segundo a duração do sono noturno. A coleta de dados foi realizada por meio de questionário de caracterização, Exame Cognitivo de Addenbrooke – Revisado (ACE-R) e Índice de Qualidade do Sono de Pittsburgh (PSQI). Foram feitas análises estatísticas descritivas, comparativas e correlacionais.

**Resultados:**

os idosos obtiveram média de 61,94 pontos no ACE-R e 55,1% apresentavam boa qualidade de sono. As análises comparativas evidenciaram diferenças entre os grupos apenas no domínio cognitivo fluência verbal (p=0,018). As análises *post-hoc* apontaram que idosos que dormiam maior número de horas, em média 8,85 horas (Q_1_), tinham escores inferiores quando comparados aos idosos que dormiam em média 6,11 horas (Q_3_) (p=0,004) e 4,52 horas (Q_4_) (p=0,045). O modelo ajustado com aplicação do método *stepwise* apontou relação das variáveis independentes escolaridade e duração do sono com o domínio fluência verbal.

**Conclusão:**

conclui-se que a duração do sono tem relação com o domínio cognitivo fluência verbal.

## Abstract (es)

**Objetivo:**

analizar la relación entre la duración del sueño nocturno autoinformado y el rendimiento cognitivo de los adultos mayores.

**Método:**

la muestra estuvo conformada por 156 ancianos inscritos en Unidades de Salud de la Familia (USF) de una ciudad de São Paulo, divididos en cuartiles según la duración del sueño nocturno. La recolección de datos se realizó mediante un cuestionario de caracterización, el Examen cognitivo revisado de Addenbrooke (ACE-R) y el Índice de calidad del sueño de Pittsburgh (PSQI). Se realizaron análisis estadísticos descriptivos, comparativos y correlacionales.

**Resultados:**

los ancianos tuvieron una media de 61,94 puntos en el ACE-R y el 55,1% tuvo buena calidad del sueño. Los análisis comparativos mostraron diferencias entre los grupos solo en el dominio cognitivo de la fluidez verbal (p=0,018). Los análisis *post-hoc* mostraron que las personas mayores que durmieron más horas, en promedio 8,85 horas (Q1), tuvieron puntuaciones más bajas en comparación con las personas mayores que durmieron en promedio 6,11 horas (Q3) (p=0,004) y 4,52 horas (Q4) (p=0,045). El modelo ajustado con la aplicación del método *stepwise* mostró una relación entre las variables independientes educación y duración del sueño con el dominio fluidez verbal.

**Conclusión:**

se concluye que la duración del sueño está relacionada con el dominio cognitivo de la fluidez verbal.

## Introduction

In the world context, aging has stood out as a phenomenon. In 2017, the United Nations (UN) reported that the world population was 7.6 billion inhabitants and that, of these, 13% were 60 years old or older. Estimates indicate that this proportion will increase to 21% in 2050 and to 28% in 2100^(^ 1 ^)^. The occurrence of chronic non-communicable diseases (NCDs) and the set of changes common to aging can lead to a reduction in functional and cognitive capacity^(^ 2 ^)^. Concomitantly, sleep disorders stand out as a frequent complaint in the older adult population^(^ 3 ^)^.

Sleep is a physiological condition of brain activity, natural and periodic, described by the literature as a period of reversible loss of consciousness, with reduced sensitivity, homeostatic regulation, motor and sensory functioning, being a universal need that provides well-being, physical and mental rest, with energy recovery for the execution of physical and mental tasks^(^ 4 ^-^ 5 ^)^. Made up of five stages or phases, two fundamental patterns are observed: NREM (Non-Rapid Eye Movement – non-REM) sleep and REM (Rapid Eye Movement – REM) sleep. NREM sleep consists of four stages in gradual depth, stages I, II, III and IV, and is characterized by the absence of rapid eye movements. REM sleep is distinguished by the presence of rapid eye movements, being a deep stage related to the difficulty of awakening. In this phase, the electroencephalographic pattern is similar to the state of wakefulness with eyes open and of stage 1 NREM sleep^(^ 6 ^)^.

Poor quality sleep that is out of sync with the circadian rhythm is a common complaint among older adults. The fact worsens among older adults with cognitive impairment and comorbidities. However, strategies aimed at improving sleep quality can contribute not only to improving sleep, but also to the cognitive function among the older adults^(^ 7 ^)^. Non-pharmacological strategies, such as interventions with earplugs, eye masks, musical productions, muscle relaxation, postural training, meditation, relaxation and educational activities, can be conducted by several professionals, including nurses, and contribute to improving sleep quality^(^ 8 ^-^ 9 ^)^.

Throughout life, changes in pattern, architecture, circadian rhythm and wakefulness occur. Environmental factors, emotional aspects, pain, diseases and decreased melatonin production contribute to complaints related to the quantity and quality of sleep^(^ 6 ^-^ 10 ^)^. According to the *National Sleep Foundation’s*, in a systematic review, adequate sleep duration for newborns is 14 to 17 hours of sleep; for infants, 12 to 15 hours; for children, 10 to 14 hours; for adolescents, 8 to 10 hours; for adults, 7 to 9 hours and, for older adults, 7 to 8 hours. The experts pointed out that variations beyond the indicated range may or may not reveal sleep and/or health problems^(^ 11 ^)^.

In developing countries, nearly 37.7% of the older adult population have complaints related to sleep. A number of studies associate poor sleep quality with some frailty criteria, such as decreased muscle strength, slow locomotion and difficulty getting up from a chair without assistance. In addition, sleep disorders (SDs) in older adults are related to cognitive decline, tiredness, stress and lack of attention, with greater incidence on the female gender and in individuals with mood and anxiety disorders^(^ 12 ^-^ 14 ^)^.

A study carried out in 2012 with an elderly African population concluded that complaints related to sleep and its duration are factors potentially related to health, sociodemographic factors and lifestyle^(^ 15 ^)^. A review of sleep duration and its relationship to cognition in older adults mentioned sleep duration as an indicator of circadian rhythm and identified 32 studies with an association between sleep duration and cognition. Of these, 31% (n=20) indicated an association between short sleep duration and worse cognitive function in older adults^(^ 16 ^)^.

Cognition corresponds to a set of performances and processing of intellectual information, attributed by skills such as perception, memory, attention, reasoning, planning, executive function and decision-making. During aging, the cognitive changes that appear can be explained by changes that occur in the central nervous system, culminating in weight loss, slow and progressive, rendering the central nervous system incapable of making repairs to acquired morphological changes^(^ 17 ^)^.

The decline and stability of different cognitive functions, throughout the aging process, are affected by individual differences that comprise sociodemographic, genetic, lifestyle and physical health aspects. Mental capacity worsens, with numerical capacity standing out, when the individual reaches 80 years of age, followed by speed of perception and reduction in the speed of information processing^(^ 18 ^-^ 19 ^)^. Regarding executive functions, the older adults have greater difficulty in processing and elaborating adapted actions, in starting tasks, estimating time, switching from one task to another, controlling impulses, and planning and executing a task chronologically^(^ 20 ^)^.

Diverse evidence points out that changes in sleep architecture in old age increase the risk for changes in circadian rhythm, medical and psychiatric disorders, use of medications and a probable combination of these factors^(^ 21 ^)^. A study carried out in Chubu, Japan, with the objective of demonstrating an association between poor sleep quality (and/or insufficient sleep) and worse cognitive performance, especially in attention, in an elderly community, made evaluations using the Continuous Performance Test (CPT) and the Number-back Test and concluded that sleep can play an important role in the differences in cognitive performance in older adults^(^ 22 ^)^.

Currently, much research has been done in relation to sleep and cognitive processes due to the importance of sleep for memory, attention, reasoning, psychomotor alertness and visuospatial skills^(^ 23 ^)^. Several clinical situations in which sleep deprivation occurs are associated with cognitive and memory deficit^(^ 24 ^)^.

The literature points out that the aging process causes changes in the sleep/wake pattern of the older adults, thus impairing their cognitive abilities, especially executive functioning. Cognitive changes and decreased strength and balance increase the risk of falls^(^ 25 ^-^ 26 ^)^. Falls can have different etiologies, but the occurrences are more frequent with advancing age^(^ 27 ^)^. The sensation of non-restorative sleep can also be associated with a poor perception of health and dissatisfaction with life^(^ 28 ^-^ 29 ^)^.

Investigating aspects related to sleep from the perspective of the older adults reflects a search for comprehensive care. Considering sleep, its quality and duration as fundamental aspects for the well-being, balance, good functioning and maintenance of the organism, and the importance described in the literature with influence on cognition, the present study aims to analyze the relationship between duration of self-reported night sleep and the cognitive performance of the community older adults. However, it is hypothesized that older adults who sleep more hours have better cognitive performance.

## Method

This was a cross-sectional and descriptive study, based on the quantitative method of research. It was conducted in a municipality in the inland of the state of São Paulo (Brazil), in the period from June 2016 to January 2018.

From the total number of older adults assisted in the Family Health Units (FHUs) of the city (n=5,130), it was calculated that 150 older adults would constitute a sample with a 95% confidence level and a margin of error of 7.9%. The non-probabilistic sample consisted of older adults aged 60 or over who resided and were registered in the areas covered by the FHU. A total of 156 older adults who met the inclusion criteria participated in the study. The older adults who had hearing problems such as deafness and/or language problems such as aphasia, dysphemia or apraxia of speech that prevented the application of the instruments were excluded.

From lists provided by the FHU, the researchers visited the older adults at their homes to verify and confirm the inclusion and exclusion criteria. When completed, the older adults were invited to participate in the research. After accepting and signing the Free and Informed Consent Form, sociodemographic characterization information and data related to the variables of interest in this study: cognition and sleep were collected. To this end, the following instruments were used:

(a) Questionnaire of sociodemographic characterization of the older adult: it consisted of a form to collect data on gender (male/female), age (in years old), marital status (married/partner, single, widowed, divorced/separated/divorced), schooling (in years), retirement (yes/no), and individual and family income (in reais).
(b) Exam Addenbrooke’s Cognitive – Revised (ACE-R): it was developed in 2006, and translated and validated for Brazilian Portuguese in 2007. It consists of a brief cognitive assessment battery, which ranges from 0 to 100 points and has five domains, each with a specific score, namely: Attention and Guidance (total score of 18 points); Memory (total score of 26 points); Fluency (total score of 14 points); Language (total score of 26 points) and Visuospatial (total score of 16 points)^(^ 30 ^-^ 31 ^)^. The total ACE-R scores were used for the analysis of this study, as well as the domain scores.
(c) Pittsburgh Sleep Quality Score: Elaborated in 1989, and translated, adapted and validated for the Brazilian context in 2008, it is used to assess sleep quality in the last month. Consisting of 19 self-reporting questions, grouped into seven components, namely: subjective sleep quality, sleep latency, sleep duration, usual sleep efficiency, sleep disorders, use of sleeping medication, and daytime dysfunction. The components add up to an overall score ranging from 0 to 21 points. From this score, the quality of sleep can be classified into: good (0 to 4 points); poor 5 to 10 points) or presence of disorder (above 10 points)^(^ 32 ^-^ 33 ^)^. Question 4 of the instrument was used to compose the groups for comparative analyses (“During the past month, how many hours of sleep *per* night did you sleep?”).

The data obtained were entered into a bank in the *Statistical Package for Social Sciences* (SPSS) for Windows program, version 19.0, to perform the following: descriptive analyses, Pearson’s Chi-square test, Kruskal-Wallis test, Mann-Whitney and Linear regression. In the models, cognitive performance (ACE-R and Verbal Fluency) was treated as a dependent variable and the independent variables were hours of sleep, age, gender, schooling and use of medications. For comparative analyses of cognition, the participants were divided into four groups (quartiles) according to the number of hours of self-reported night sleep. The p-value was considered as the level of statistical significance at 5% (p<0.05).

All the ethical principles were respected, and the project was approved by the Research Ethics Committee of the Federal University of São Carlos and by the Municipal Health Secretariat of São Carlos.

## Results

The older adults in the sample were mostly female (n=125; 80.1%), with a mean age of 70.4 years old (±6.8), married or living with a partner (n=84; 53.8%), with a mean of 3.65 (±3.3) years of study. Regarding retirement, 128 (82.1%) were retired with mean incomes of R$ 1,117.87 (USD 353.75) and of R$ 2,007.97(USD 635.43), individual and family, respectively. For reference purposes, the minimum wage values in force at the beginning and end of data collection (June 2016 and January 2018) were R$ 880.00 and R$ 954.00, respectively The amounts were converted into dollars on 01/31/2018, end of collection, with the value of USD 1 = R$ 3.16 available on the Central Bank of Brazil website and, when converted, they were USD 278.48 and USD 301.89.

The detailed characterization of sleep quality by domain is shown in Table 1. Regarding the total PSQI scores, 55.1% (n=86) of the older adults presented good sleep quality. However, 41.7% (n=65) of the older adults had poor sleep quality and 3.2% (n=5) presented scores that showed sleep disorders.

**Table 1:** Descriptive analyses related to the sleep of the older adults (n=156) according to the domains of the Pittsburgh Sleep Quality Index. São Carlos, SP, Brasil, 2018

Domain	N	%	Mean (SD*)	Median	Variation (min-max)
**Subjective sleep quality**					
Very good	45	28.9			
Good	76	48.7			
Poor	25	16.0			
Very poor	10	6.4			
**Sleep latency (minutes)**			28.2 (±35.7)	10.0	1-180
< or = 15 minutes	81	51.9			
16-30 minutes	31	19.9			
31-60 minutes	25	16.0			
60+ minutes	19	12.2			
**Sleep duration (hours)**			7.04 (±1.84)	7.00	3-12 hours
More than 7 hours	93	59.6			
6 to 7 hours	29	18.6			
5 to 6 hours	11	7.1			
Less than 5 hours	23	14.7			
**Usual sleep efficiency (%)**			88.74 (±18.15)	96.57	36.14-150.00
> 85%	107	68.6			
75 to 84%	13	8.3			
65 to 74%	12	7.7			
< 65%	24	15.4			
**Sleep disorder**					
Absence of disorder	27	17.3			
Mild disorder	114	73.1			
Moderate disorder	15	9.6			
Severe disorder	--	--			
**Use of sleep medications**					
Not once	120	76.9			
Less than 1 time a week	5	3.2			
1 to 2 times a week	2	1.3			
3 times a week	28	18.0			
Did not answer	1	0.6			
**Daytime dysfunction**					
Absence of daytime dysfunction	112	71.8			
Mild daytime dysfunction	34	21.8			
Moderate daytime dysfunction	6	3.8			
Severe daytime dysfunction	3	2.0			
Did not answer	1	0.6			

* SD = Standard deviation

According to the ACE-R domains, the older adults obtained 13.65 points (Mn=13.00; SD=2.62) in the attention and orientation domain; 14.28 points (Mn=14.00; SD=5.90) in the memory domain; 5.74 points (Mn=5.74; SD=2.97) in verbal fluency; 18.08 points (Mn=18.00; SD=5.39) in language; and 10.20 points (Mn=10.00; SD=3.50) in visuospatial skills.

**Figure 1:**
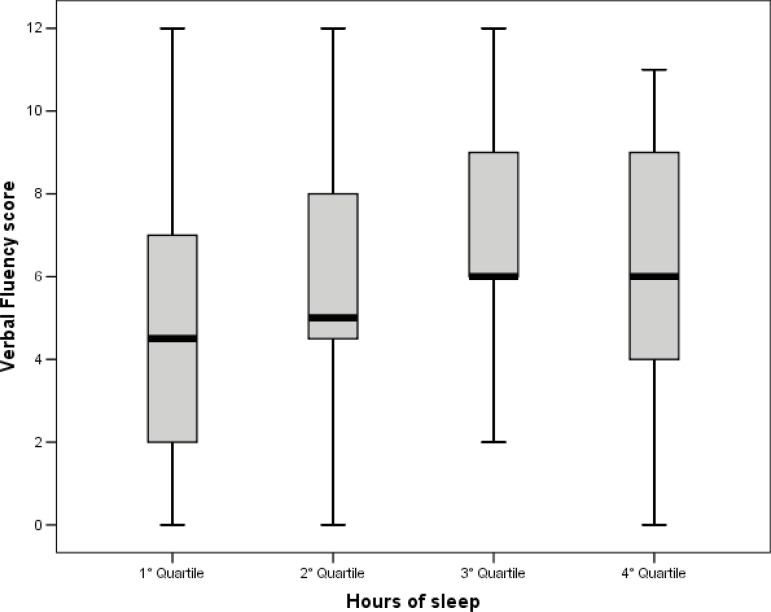
Comparison of the Verbal Fluency score per quartile of hours of sleep in the older adults (n=156). São Carlos, SP, Brazil, 2018

Table 2 presents the description and comparison of the ACE-R domains in relation to the hours slept per night (quartiles). As for the cognition domains, it is noted that, in the attention/orientation domain, there were no significant differences, as well as in the domain of memory, language and visuospatial skills. Likewise, the data did not show differences between the groups in the total ACE-R scores. However, comparative analyses showed differences between the groups in the domain of verbal fluency (*X* ^2^=10.060; DoF=3; p=0.018) (Table 2 and Figure 1).

**Table 2:** Descriptive and comparative analyses of the cognition domains according to the number of hours slept by the older adults (n=156). São Carlos, SP, Brasil, 2018

Quartiles by hours of sleep	1^st^ Quartile (n=62) M=8.8h Q2=8.5h	2^nd^ Quartile (n=31) M=7.1h Q2=7.0h	3^rd^ Quartile (n=29) M=6.1h Q2=6.0h	4^th^ Quartile (n=34) M=4.5h Q2=5.0h		Comparative Analyses*	
**Cognitive** **Domains**						X^2^	p
**Attention**/ **Guidance**						6.490	0.090
Mean	13.18	14.06	14.52	13.38	13.65		
Median	13.00	14.00	15.00	13.00	13.00		
SD	2.62	2.88	2.44	2.34	2.62		
Memory						4.810	0.186
Mean	13.06	14.90	15.86	14.59	14.28		
Median	13.00	14.00	16.00	13.00	14.00		
SD	5.76	6.15	5.47	6.07	5.90		
**Verbal Fluency**						10.060	0.018
Mean	4.92	5.84	6.90	6.15	5.74		
Median	4.50	5.00	6.00	6.00	6.00		
SD	3.15	2.89	2.48	2.74	2.97		
**Language**						4.419	0.220
Mean	16.92	18.71	19.17	18.68	18.08		
Median	17.00	21.00	21.00	18.50	18.00		
SD	5.53	5.43	5.14	5.14	5.40		
**Visuospatial** **skills**						4.523	0.210
Mean	9.53	10.77	11.17	10.06	10.20		
Median	9.00	11.00	11.00	10.00	10.00		
SD	3.67	3.40	2.79	3.68	3.50		
**ACE-R Total^†^**						6.770	0.080
Mean	57.61	64.29	67.62	62.85	61.94		
Median	58.00	62.00	66.00	61.00	62.00		
SD	17.57	18.08	14.84	17.04	17.3		

* Kruskal-Wallis test;

† Addenbrooke's Cognitive Exam - Revised version

It is noteworthy that, for greater control in relation to biases described in the literature for the cognition dependent variable, the groups were compared to verify pairing in relation to age, schooling, individual and family income, gender, daytime dysfunction, daytime naps and use of sleep medication and there was no significant difference between the groups in relation to age, schooling, individual and family income, gender, daytime dysfunction, daytime naps and use of sleep medication (p>0.05 in all the analyses). The groups were similar in relation to these variables and there was an equal distribution of conditions in all groups in the case of the categorical variables (Table 2).

The *post-hoc* comparative analyses showed that older adults who slept more hours, a mean of 8.85 hours (Q_1_), had lower scores in the verbal fluency domain when compared to those who slept a mean of 6.11 hours and of 4.52 hours (Figure 1).

From the results found in the comparative analyses, multiple linear regression analysis was continued only for verbal fluency, which showed a significant difference between the sleep quartiles and the total ACE-R scores that refer to the general objective of this research.

For the verbal fluency domain of ACE-R, the correlational analyses pointed to the absence of multicollinearity (rho>0.80) and that probably the important variables for the model were schooling (rho=0.464; p=0.000) and sleeping hours (rho=-0.236; p=0.003). The analysis of variance (ANOVA) showed no evidence to reject the model [F^(^ 5.149)=9.502, p=0.000, R^2^=0.242]. However, the sleeping hours, gender, age and use of medication variables could have a regression coefficient equal to zero (Table 3).

**Table 3:** Linear regression analysis, stepwise model, according to the identification of factors associated with verbal fluency and hours of sleep in the older adults (n=156). São Carlos, SP, Brasil, 2018

	Non-standardized coefficients					95% confidence interval for β	
	β	Standard Error	Β	t	p-value	Lower limit	Upper limit
**Constant**	6.237	2.680		2.328	0.021	0.942	11.532
**Hours slept**	-0.274	0.119	-0.171	-2.303	0.023	-0.510	-0.039
**Age**	0.004	0.033	0.009	0.127	0.899	-0.060	0.069
**Schooling in years**	0.397	0.069	0.440	5.752	0.000	0.261	0.534
Gender*	-0.170	0.575	-0.023	-0.295	0.768	-1.307	0.967
**Use of medication^†^**	0.021	0.185	0.008	0.111	0.912	-0.346	0.387

* Gender = (1) Male;

† Use of sleep medications = (0) Does not use

In view of these data, we proceeded to an adjusted model using the stepwise method to define which variables would be really significant for the model. The analysis of variance (ANOVA) indicated that the adjusted model should not be rejected [F(2.152)= 24.168, p=0.000, R2=0.241] and the schooling (β=0.444; t= 6,242; p=0.000) and hours of sleep (β=-0.165; t=-2.319; p=0.022) independent variables remained in the model.

For the total ACE-R scores, the correlational analyses showed that probably the important variables for the model were schooling (p=0.000), age (p= 0.001) and hours slept (p=0.029), respectively; there was no multicollinearity (rho>0.80). Thus, it was performed with multiple linear regression analysis. The analysis of variance (ANOVA) showed no evidence for rejection of the model [F^(^ 5.149)=19.3888, p=0.000, R^2^=0.394]. However, the hours slept, gender, age and use of medication variables could have a regression coefficient equal to zero (Table 4).

**Table 4:** Linear regression analysis, stepwise model, according to the identification of factors associated with the older adults' cognition (n=156). São Carlos, SP, Brasil, 2018

	Non-standardized coefficients		Standardized coefficient			95% confidence interval for β	
	Β	Standard Error	Β	t	p-value	Lower limit	Upper limit
**Constant**	80.377	13.934		5.769	0.000	52.844	107.910
**Hours slept**	-0.960	0.619	-0.103	-1.550	0.123	-2.183	0.264
**Age**	-0.288	0.170	-0.113	-1.699	0.091	-0.623	0.047
**Schooling in years**	2.949	0.359	0.562	8.210	0.000	2.239	3.659
Gender*	-1.116	2.992	-0.026	-0.373	0.710	-7.028	4.795
**Use of medication^†^**	-0.043	0.964	-0.003	-0.045	0.964	-1.948	1.861

* Gender = (1) Male;

† Use of sleep medications = (0) Does not use

In this way, we proceeded with the analyses for an adjusted model using the stepwise method to define which variables would be really significant for the model. The analysis of variance (ANOVA) indicated that the adjusted model should not be rejected [F^(^ 1.153)=90.099, p=0.000, R^2^=0.371]. However, the stepwise method removed the hours slept, gender, age and use of medications variables, only considering schooling as a predictor of cognition (β=0.609, t=9.492, p=0.000).

## Discussion

The literature describes the community older adults assisted in the FHUs with demographic characteristics similar to those found in this study^(^ 34 ^-^ 38 ^)^. The majority proportion of females among the older adults is a consequence of the higher mortality rate in men at all stages of the life cycle, which results in an unbalanced and expressive proportion in the more advanced life phases. This phenomenon is known as the feminization of old age^(^ 39 ^)^.

In 2018, in order to assess factors associated with happiness in a sample of longer-lived older adults, 263 older adults were interviewed with a mean age of 70.2 years old and 3.1 years of schooling^(^ 40 ^)^. The older adults had a mean of 3.6 years of study ranging from zero to 15 years of schooling. According to IBGE data, in 2016 the illiteracy rate in the country was estimated at 7.2%, with 11.8 million illiterate individuals. Among the older adult population, it is noteworthy that 81.8% of the Brazilians older adults present a mean of 3.7 years of study, that is, they only attended elementary school^(^ 41 ^)^. With regard to retirement and individual income, there is an important representation of the older adult for the support of the family, which does not differ from data of other studies^(^ 27 ^,^ 37 ^,^ 42).

Regarding the PSQI domains, the older adults perceived their own quality of sleep as good or very good, took a certain amount of time to fall asleep, had more than seven hours of sleep per night, had a habitual sleep efficiency considered normal, did not have sleep disorders or daytime dysfunction, and did not use sleep medications. A study developed with 100 older adults seen at a geriatric outpatient clinic of a University Hospital in João Pessoa-PB, showed positive results that are close to those found in this research. Eighty eight percent of the older adults had never used sleep medications, followed by 0.4% who reported that the use of medications for this purpose was very rare^(^ 43 ^)^.

The analysis of sleep quality reflected good quality. Although it is a frequent complaint among the older adults, a study of myths and truths about aging pointed out that the findings in this study contribute to the literature. Nearly 60% of the older adults believed that longer-lived people did not feel less sleep^(^ 44 ^)^. However, another study found that 46% of the older adults presented very altered sleep and had a negative self-perception of sleep quality (p<0.001); when asked about their self-evaluation of sleep, 57% reported good sleep quality, followed by 20% of the older adults classifying it as poor^(^ 43 ^)^. A similar phenomenon was observed in Yilan, Taiwan, which assessed 2,622 older adults living in the community. Of these, only 1,011 ^(^ 38.6%) presented poor sleep quality according to PSQI and, when present, they were longer-lived older adults (p=0.04)^(^ 45 ^)^.

As for the number of hours slept, the *National Sleep Foundation’s* establishes as suitable for older adults from 7 to 8 hours of sleep; however, it does not expose a specific number of hours that are inadequate, placing individual diversity as an important factor in the number of hours needed to maintain health and well-being^(^ 11 ^)^.

In 2018, participants in two studies, the ENRICA - Study on Nutrition and Cardiovascular Risk Factors in Spain (2012-2015, n=1,773) and in the ELSA - English Longitudinal Study of Aging cohort (waves 4 and 6, n=4,885), individuals aged over 60 years old were evaluated. According to the results of the study, the older adults had a mean of 7.0 hours of sleep and presented poor sleep quality both in Spain and in England^(^ 46 ^)^. In Canada, a research carried study out by the Western University, by means of an online platform of the Cambridge Brain Science, investigated the dissociable effects of self-reported daily sleep duration in 10,314 participants and noted that age was associated with less sleep, as well as with sleeping less. The respondents reported sleeping a mean of 6.42 hours a night in the last month^(^ 47 ^)^. A research study carried out with Nigerian older adult women aimed at identifying the risk of impaired sleep in women who lived in urban areas. Of the 428 interviewees, one hundred and seventeen ^(^ 27.3%) obtained a mean overall PSQI score of 4.4 (SD=3.1), indicating the presence of sleep disorders. The mean sleep duration of the sample was 7.0 hours (SD=1.4 hours), with 2.8% reporting sleeping a mean of less than 5 hours a night^(^ 48 ^)^. The sleep data of the older adults such as good quality, good efficiency, and mean of 7.04 hours of sleep per night, corroborate with descriptions present in the literature^(^ 43 ^-^ 46 ^,^ 48 ^-^ 49).

Sleep is a necessary physiological condition for the human body and its deprivation implies possible cognitive changes. In a prospective observational study conducted in 2014, with the objective of identifying an association between circadian rhythm activity and cognitive function, with regard to global cognition, verbal and working memory and executive function, 1,287 women from the community with a mean age of 82.8 years old were the study object. When comparing cognitive performance with baseline actigraphy using covariance analyses adjusted for a number of health factors and comorbidities, they concluded that the weaker/interrupted rhythmic patterns of circadian activity are associated with worse cognitive performance. However, although the study presents relevant points and control of multiple variables for cognition and sleep, the authors pointed out limitations regarding gender, since it is an exclusively female sample, and it is not possible to generalize the finding. There were no detailed tests of cognition at the beginning of the study; however, the authors suggest believing in the confidence of their results, since the analyses were consistent for the participants’ cognitive function in four models adjusted to several factors of the sample; they still suggest that circadian rhythm activity, in the future, can be a biomarker for developing strategies and interventions aimed at sleep and improving healthy aging^(^ 50 ^)^.

Another study carried out in 2012 in Sichuan, located in southeastern China, was conducted with the aim of associating quality of sleep and cognitive function among long-lived older adults over 90 years old. Of the 660 participants with a mean age of 93.52 years old, 69 were centenarians and 444 were women. Regarding sleep quality, 58.4% presented good sleep, followed by 19.4% reasonably good and 22.2% poor. The older adults who had good sleep quality were younger (p=0.016). The older adults who presented poor sleep quality had lower scores on the cognitive assessment score (p=0.007). The authors concluded that there is an association between cognitive impairment and sleep quality in long-lived individuals^(^ 51 ^)^.

The researchers investigated women (n=2,932) with a mean age of 83.5 years old, aiming to associate the objective measurement of sleep by means of an actigraph (sensitive device for detecting movement and light) and cognition, and found that women who had a sleep efficiency of less than 70% and greater sleep latency had a higher risk of cognitive impairment. However, they found no significant relationship for the total sleep time and cognition^(^ 52 ^)^.

On sleep duration, in a study with rural residents aged 40 and over, they interviewed 3,840 South Africans, 44.1% of whom were over 50 years old. Of these, 8.3% presented cognitive impairment. The authors stated that sleep duration is potentially related to sociodemographic conditions, the subjects’ lifestyle, depression and acute myocardial infarction, and did not observe any association between sleep duration and other chronic health conditions. Finally, the authors suggest the need for longitudinal studies in order to better understand possible associations^(^ 53 ^)^.

An American study by the National Institute on Aging Grant, carried out with 144 older adults over 90 years of age, with the aim of assessing the quantity and quality of sleep in relation to hippocampal cognition, concluded that sleep duration greater than eight hours was associated with lower scores on global cognition, memory and executive function tests, concluding that a very prolonged sleep duration is a risk factor for cognitive worsening in older adults^(^ 54 ^)^. Another study carried out in 2006 aimed to investigate the association between sleep duration, snoring and difficulty sleeping with the cognitive function in community women, aged between 70 and 81 years old. The analyses showed that individuals who slept between six and eight hours per night obtained better cognitive scores for the verbal fluency category than those who slept five hours or less and nine hours or more per night. However, the analyses did not remain statistically significant after adjusting the confounding variables^(^ 55 ^)^.

In 2017, researchers subjected 41 older adults to cognitive training and sleep hygiene. The older adults constituted four groups: control group, cognitive training group, sleep hygiene group, and hygiene training group. The results showed that the latter group achieved an improvement in cognitive flexibility tasks, problem solving, verbal fluency, attention, and episodic memory. In addition, they obtained gains in sleep quality and a reduction in terms of excessive daytime sleepiness. Thus, they concluded that cognitive training and sleep interventions were successful strategies for improving cognitive performance, as well as for the quality of sleep in the older adults^(^ 56 ^)^.

A prospective study conducted with 15,385 nurses aged 70 years old and over in 2014, with the objective of evaluating associations between sleep duration, change in sleep duration over time and cognition, concluded that individuals who slept more presented a significant association with worse cognitive performance, both in total cognition scores and for the verbal fluency domain^(^ 57 ^)^. Another study with the purpose of observing the relationship between total sleep time and cognitive function in adult life of young and older adult participants in a community, showed that short or very long total sleep time was associated with worse working memory and verbal fluency, especially in younger older adults with a mean age of 62.68 years old^(^ 58 ^)^.

In order to determine the association between actigraphic sleep duration and fragmentation with cognition in older adult women, 782 women with a mean age of 87.4 years old with different cognitive states were evaluated. The sample was divided by tertile of sleep time and awakening after sleep, with little significant association in the adjusted analyses. However, significant adjusted associations of total sleep time with impaired cognitive performance and waking up after sleep with impaired memory, semantic fluency and digit range^(^ 59 ^)^ were observed.

A study conducted in 2019 described that changes in sleep duration had significant associations with greater cognitive decline among the elderly. The authors concluding that sleep durations between 6 and 9 hours and increased duration were negatively associated with certain aspects of cognition, such as in backward digit span cognitive performance and in cognitive tests for verbal fluency^(^ 60 ^)^.

In general, the results presented in this research corroborate data recently published in the academic environment^(^ 52 ^-^ 55 ^,^ 57 ^-^ 60). Cognitive performance has no linear relationship with sleep duration. Therefore, extreme conditions, of very long and very short sleepers, do not positively influence cognitive ability. The specific condition of language impairment in long sleepers found in the present result was also described only in two other international studies^(^ 58 ^,^ 60 ^)^. Therefore, future research studies with greater control of variables related to this domain would be important to elucidate these notes.

It is noteworthy that this research was carried out with a small non-probabilistic sample, with data from residents in areas covered by the FHUs, which can be considered a limitation of the study, and even if a sample calculation was performed, the municipality does not have high coverage. coverage by FHU, not reaching 30% of the population. Limitations like this could be overcome with further research studies in other locations with robust probabilistic samples. Despite the limitations, the findings of this study bring important advances on the theme for both the health and nursing areas. The fact that the older adult is a long sleeper can be indicative of cognitive decline. The results indicate that the best for cognitive health are intermediate periods of sleep, being the extreme indicators of alertness for health professionals. In addition, international studies contemplate older adults with a demographic profile different from the Brazilian reality. In the national context, this is the only study that relates language impairment to long sleep duration.

## Conclusion

The present study allowed describing sociodemographic characteristics, quality and duration of sleep, as well as the cognitive status of a sample of older adults seen in the FHUs of the city. Sleep quality was considered good for most of the older adults with a mean sleep duration per night. The older adults showed good cognitive performance regarding the total ACE-R scores.

No relationship was found between the duration of self-reported nighttime sleep and general cognitive performance. Therefore, it is concluded that older adults who sleep more hours do not have better cognitive performance, that is, long sleeping does not mean a favorable condition for cognition. However, sleep duration was related to the verbal fluency domain, with older adults who slept more hours having lower scores in the domain.

However, the results bring important information that may support future research studies and are part of the set of research that seeks to assist with data that can support the planning of multidisciplinary actions and contribute to Nursing in order to develop care strategies, with education in health, sleep hygiene measures and cognitive workshops, aimed at improving care for the older adults with a view to promoting the health and quality of life of this population within the scope of Primary Care.
